# On Some Points Connected with Cholera

**Published:** 1889-09-07

**Authors:** 


### On Some Points Connected with Cholera.

*(Concluded from page 338.)*

### COMMON SYMPTOMS.

Another predisposing cause is the use of purgative medicine, especially saline purgative medicine, during a cholera epidemic. At such periods purgatives should, if possible, not be used, and the safest is castor oil. And if used, they should never be taken at night. As regards class, men are proportionally more liable than women, except pregnant women, who are specially liable. Hill people descending to the plains in India are specially liable; so are children, but not infants. Persons suffering from cholera are rarely attacked again during the same epidemic, but a previous attack confers no exemption in future epidemics, a fact altogether opposed to the alleged value of the Italian Ferran's innoculations for the prevention of the disease.

Diagnosis of Cholera.—There are symptoms arising from other causes which may be mistaken for cholera. These causes are principally diarrhœa, arsenic poisoning, impure water, stale fruit, stale fish, fungi mistaken for mushrooms, the fruit of the *luffa echinata*, impure milk, impure cheese, and colic.

Diarrhœa may be attended with cramps and all the early symptoms of cholera, excepting rice-water stools and stoppage of urine, which are absent in mere diarrhœa. But cholera often commences as diarrhœa, and gradually develops into cholera. It is often impossible to define with correctness whether the attack should be called cholera or diarrhœa. Summer diarrhœa is induced; if not by heat, at least during the period of the greatest heat. Summer diarrhœa is often associated with cramps, and the diarrhœa of children assumes the likeness of cholera to such an extent as to have attained the name *cholera infantum*. Moreover, when cholera prevails, diarrhœa, due evidently to similar influences, is also present. The question therefore arises, at what stage does the sufferer from diarrhœa, become the victim of cholera? To regard the case as cholera only, when two prominent symptoms occur, is scarcely consistent with the fact of so many cases commencing as diarrhœa and terminating as cholera, or with what is admitted in other diseases; diphtheria, for instance, of which there may be very mild and very severe cases. The term choleraic diarrhœa has been unadvisedly expunged from the official nomenclature, but there is no other term applicable to many cases. Mere degree is not sufficient ground for regarding a disease different in essential character. There are mild and severe degrees of influenza, scarlet fever, diphtheria, typhoid, etc., some so mild as to be scarcely recognisable. But it has become the custom to regard cholera as a disease when marked by certain signs only, and when it spreads epidemically, and to say a malady is not cholera when all these signs are not present, and when it does not spread. This view we are not prepared to endorse.

The variability of the types of numerous other diseases is fully recognised. No one would deny a particular case to be one of diphtheria, or typhoid, or measles, because the symptoms were mild, or because all the symptoms of a typical case were not presented, and it is only reasonable that the same breadth of view should be accorded to cholera. Then it would be recognised as what it really is—viz., a disease occurring at all times and in all countries, although not with the same degree of violence. Then we should recognise in summer diarrhœa a mild phase of cholera.

Arsenic poison is characterised by vomiting and purging, but there is not ordinarily stoppage of urine, although this symptom has occurred. Also there are not rice-water stools, but mucus streaked with blood. Moreover, in arsenic poisoning pain in the throat precedes vomiting; if it occurs in cholera, it is a result of the vomiting.

Impure water, especially brackish water containing mineral salts, as so frequently met with in Western India, may excite griping, purging, and vomiting, but the cholera characteristics of rice-water stools and stoppage of urine are absent. Similar remarks apply to the symptoms excited by stale fruit. But vomiting, purging, and suppression of urine have been known to follow eating poisonous fungi, although no rice-water stools. Stale fish, and especially oysters, particularly if taken from the roots and branches of mangrove trees or if a shell has opened from some injury and decomposition commenced, sometimes excite all the symptoms of cholera. It should be recollected that a fish which is wholesome when absolutely fresh may become otherwise if kept only a few hours in a moist hot atmosphere, from the formation of a post mortem poison or ptomaine. The fruit or nut of an Indian creeping plant, the *luffa echinata* (native name, *deodagri*), is also known to excite symptoms much resembling cholera, but no rice-water stools. Impure milk, or milk in which some decomposition has taken place, may, if taken copiously, produce all the symptoms of cholera; for the milk, passing through the intestines more or less unchanged, gives rise to white fluid stools. Decomposed cheese may also produce choleraic symptoms. The symptoms of colic are so different from those of cholera that no medical man would confuse them. Yet colic has not unfrequently been mistaken for cholera by sufferers and friends.

When a case resembling cholera occurs in the absence of an epidemic it will be well to enquire into the possibility of such causes of ailment.

There is another important point, and that is the incubation of cholera. The weight of evidence tends to the belief that the incubation of cholera average three and a half days. But it may occur in a few hours or be extended to eleven days. Pettenkofer gives two and a half days to five, with an average of three. Sircar gives two to three days, with an average of a week. Ziemssen says five or six days. Bryden, as the result of the investigation of 611 cases, three to eleven days. Bain gives forty hours. Waters, of Bombay, says four days is an unusually lengthened period. Others writers mention from two to fourteen days. At the commencement of 1882, shortly after the Egyptian authorities first instituted quarantine against Bombay, the subject was brought before the Bombay Medical and Physical Society. At a full meeting, the members present being both native and European practitioners, it was voted unanimously that the quarantine regulations against Bombay were useless and unnecessary. This conclusion was arrived at chiefly on the grounds of the incubation of cholera being limited to ten days, a period too short to admit of the passage of a vessel from Bombay to Suez. It would be impossible to assemble a body of gentlemen having more experience of cholera than those taking part in the discussion, and their deliberately expressed opinion was doubtless calculated to strengthen the position of the British authorities in requiring the removal of the vexatious system of cholera quarantine imposed at Suez under the idea of the Continental delegates that cholera was always promulgated by direct human intercourse. Quarantine can only be supported on the view of cholera being always communicated by human intercourse, the facts showing that although it may be so communicated, still that it is not even most frequently so communicated. Quarantine in such cases is not only a waste of time, money, and energy, but is also often a cause of much unnecessary suffering to the sick and danger to the healthy. We agree with Sir Joseph Fayrer, who remarked that we might as well endeavour to keep out a flight of locusts as cholera by quarantine.

